# Targeting glutaminolysis in chondrosarcoma in context of the *IDH1/2* mutation

**DOI:** 10.1038/s41416-018-0050-9

**Published:** 2018-03-26

**Authors:** Elisabeth F. P. Peterse, Bertine Niessen, Ruben D. Addie, Yvonne de Jong, Arjen H. G. Cleven, Alwine B. Kruisselbrink, Brendy E. W. M. van den Akker, Remco J. Molenaar, Anne-Marie Cleton-Jansen, Judith V. M. G. Bovée

**Affiliations:** 1 Department of Pathology, Leiden University Medical Center, Leiden, The Netherlands; 2 Department of Medical Biology, Academic Medical Center, University of Amsterdam, Amsterdam, The Netherlands

**Keywords:** Bone cancer, Sarcoma

## Abstract

**Introduction:**

Chondrosarcoma is a malignant cartilage-forming bone tumour in which mutations in *IDH1* and *IDH2* frequently occur. Previous studies suggest an increased dependency on glutaminolysis in *IDH1/2* mutant cells, which resulted in clinical trials with the drugs CB-839, metformin and chloroquine. In this study, the preclinical rationale for using these drugs as a treatment for chondrosarcoma was evaluated.

**Methods:**

Expression of glutaminase was determined in 120 cartilage tumours by immunohistochemistry. Ten chondrosarcoma cell lines were treated with the metabolic compounds CB-849, metformin, phenformin (lipophilic analogue of metformin) and chloroquine.

**Results:**

A difference in glutaminase expression levels between the different tumour grades (*p* = 0.001, one-way ANOVA) was identified, with the highest expression observed in high-grade chondrosarcomas. Treatment with CB-839, metformin, phenformin or chloroquine revealed that chondrosarcoma cell lines are sensitive to glutaminolysis inhibition. Metformin and phenformin decreased mTOR activity in chondrosarcoma cells, and metformin decreased LC3B-II levels, which is counteracted by chloroquine.

**Conclusion:**

Targeting glutaminolysis with CB-839, metformin, phenformin or chloroquine is a potential therapeutic strategy for a subset of high-grade chondrosarcomas, irrespective of the presence or absence of an *IDH1/2* mutation.

## Introduction

Chondrosarcoma is the second most common primary bone malignancy in humans. It represents a heterogeneous collection of cartilage-forming tumours, which can be divided in several subtypes and histological grades.^1^ The most common subtype is conventional chondrosarcoma (85%), which arises centrally in the medulla of the bone. Conventional chondrosarcoma is histologically graded to determine treatment strategy and the patient’s prognosis. The atypical cartilaginous tumour (ACT, previously known as chondrosarcoma grade 1), accounts for 61% of cases. First-line treatment consists of curettage with local adjuvant treatment, resulting in a 5-year survival rate of 95%. Grade II (36%) and grade III (3%) chondrosarcomas have a worse 5-year survival of 86% and 58%, respectively, due to the occurrence of metastases.^1–3^ These tumours are treated with *en bloc* resection. Dedifferentiated chondrosarcoma is a highly malignant subtype with an overall survival rate of 7–24%.^4^ Mesenchymal chondrosarcoma has a 10-year survival rate between 44 and 54%.^5,6^ It is a rare aggressive subtype in which distant metastasis can be identified even after 20 years.^5–7^ Chondrosarcoma patients with inoperable disease, due to tumour location, tumour size or extensive metastatic disease benefit from a doxorubicin-based chemotherapeutic regimen, which increases the 3-year survival from 8 to 26%.^8^ As the overall efficacy of chemotherapy is limited, new treatment options are needed, which can be identified by further unravelling the essential driver genes and pathways of these tumours.

Potential driver mutations of central conventional and dedifferentiated chondrosarcoma are gain of function mutations in *isocitrate dehydrogenase 1* and *2 (IDH1* and *IDH2)*, which have been identified in 38–70% of the cases.^9,10^ Its occurrence in the benign precursors lesions (enchondromas), of which 52–87% harbour an *IDH1/2* mutation,^11,12^ further demonstrates that *IDH1/2* mutations are an early event in chondrosarcoma genesis. IDH1 and IDH2 are essential enzymes in cell metabolism, as they convert isocitrate to α-ketoglutarate (α-KG) in respectively the cytoplasm and the mitochondria. The mutant enzyme acquires the activity to convert α-KG to *D*-2-hydroxyglutarate (*D*-2-HG), an oncometabolite that competitively inhibits the α-KG dependent enzymes by the high structural similarities.^13^ Processes involved in chondrosarcoma progression make these cells independent of the mutant IDH enzymes, as treatment with AGI-5198, a specific IDH1 mutant inhibitor, did not influence the tumourigenic properties of these cells.^14^ Therefore, we propose to exploit the metabolic vulnerability caused by the *IDH1/2* mutations as therapeutic strategy for chondrosarcoma.

**Fig. 1:**
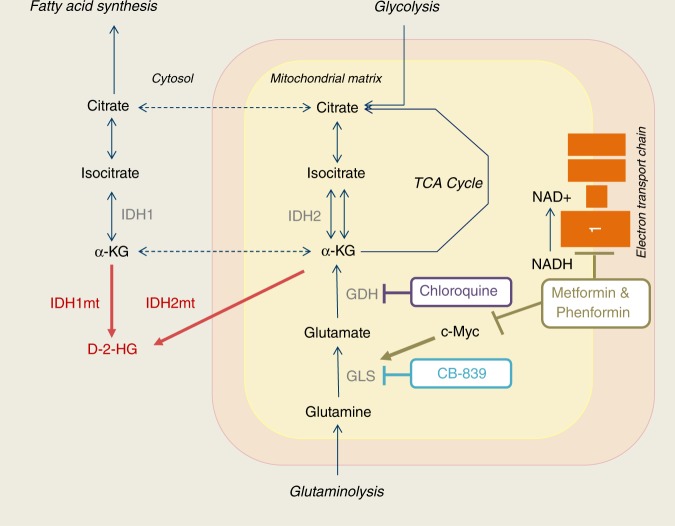
Schematic representation of glutamine metabolism and the compounds used in this study. IDH isocitrate dehydrogenase, IDHmt mutated IDH, *D*-2-HG *D*-2-hydroxyglutarate, α-KG α-ketoglutarate, GLS glutaminase, GDH glutamate dehydrogenase, NAD nicotinamide adenine dinucleotide, TCA tricarboxylic acid

*IDH1/2* mutant cells need α-KG for the production of *D*-2-HG, which can be generated via glycolysis and glutaminolysis. It has been suggested that *IDH1/2* mutated tumours depend on glutaminolysis for their α-KG supply,^15–17^ which led to two clinical trials that were recently started in *IDH1/2* mutated solid tumours, including chondrosarcomas. The first one is a phase I trial with the drug CB-839 (NCT02071862 clinicaltrials.gov), an inhibitor of glutaminase (Fig. 1). The second one is a phase IB/II trial with the drugs metformin and chloroquine (NCT02496741 clinicaltrails.gov), after which the feasibility of phenformin may be explored as an alternative to metformin in case of lack of effect of metformin.^18^ Metformin is a first-in-line drug used for the treatment of type II diabetes mellitus that inhibits gluconeogenesis in the liver. It has several effects on cellular proteins, among which it (1) activates adenosine monophosphate activated protein kinase (AMPK), thereby inhibiting the mammalian target of rapamycin (mTOR);^19^ (2) inhibits complex 1 of the electron transport chain;^20^ and (3) indirectly inhibits glutaminase, the enzyme that converts glutamine to glutamate, via c-Myc; (Fig. 1).^21,22^ Phenformin is a lipophilic analogue of metformin with similar working mechanisms, but in contrast to metformin it does not depend on solute carrier (SLC) 22A1-3 transport to get into cells;^20,23^ The anti-malaria drug chloroquine, in addition to its well-known anti-autophagy potency, is able to inhibit glutamate dehydrogenase, an enzyme converting glutamate to α-KG (Fig. 1).^24,25^

In this study, we evaluate whether there is preclinical rationale to target glutaminolysis as a treatment for chondrosarcoma by determining the expression levels of glutaminase in chondrosarcoma primary tumours and by evaluating the effect of metformin, phenformin, chloroquine and CB-839 on chondrosarcoma cells.

## MaterialS and methods

### Immunohistochemistry on tissue microarrays

Glutaminase monoclonal antibody (AB156776, Abcam) (1:400) was used for immunohistochemical stainings on previously generated and published formalin-fixed, paraffin-embedded tissue microarrays^26^ as described.^27^ Hundred and twenty cartilage tumours could be scored, consisting of 12 benign (enchondromas or osteochondromas), 56 ACTs, 36 grade II and 16 grade III tumours. Two independently operating observers used the following scoring procedure: intensity score (0 negative, 1 weak, 2 moderate, 3 strong) + percentage score (0 = 0%, 1 = 1–24%, 2 = 25–49%, 3 = 50–74% and 4 = 75–100%). Discrepancies were discussed to reach consensus. Of the central cartilage lesions that could be scored, the *IDH* mutation status was known of 54 tumours, of which 33 harboured an *IDH1* or *IDH2* mutation and 21 were wildtype.

### Statistical analysis

Statistical analysis on immunohistochemistry data was performed using Statistical Package for the Social Sciences 23 (SPSS Statistics, IBM). One-way ANOVA with the Fisher’s least significant difference (LSD) post-hoc analysis was used to compare glutaminase expression levels between different tumour grades. The difference in glutaminase protein expression between high-grade cartilage tumours (grade II and grade III cartilage tumours) and low-grade cartilage tumours (enchondromas, osteochondromas and ACT) was determined using independent-samples T test. Results were considered significant at the *α* = 0.05 level.

### Cell culture

Five *IDH1 or IDH2* mutated (JJ012,^28^ SW1353 (ATCC #HTB-94), L2975,^29^ L835^29^ and HT1080^30^) and five *IDH1/2* wildtype (CH2879,^31^ MCS170,^32^ CH3573,^33^ NDCS1^34^ and L3252b^29^) chondrosarcoma cell lines were analysed. Five of these originate from conventional chondrosarcoma (JJ012, SW1353, L835, CH2879 and CH3573), three from dedifferentiated chondrosarcoma (L2975, NDCS1 and L3252b) and one from mesenchymal chondrosarcoma (MCS170). HT1080 was originally reported as a fibrosarcoma of bone. As this is a diagnosis of exclusion and this cell line is now known to harbour an *IDH1* mutation, this tumour most probably reflects a dedifferentiated chondrosarcoma.^14^ Cells were cultured at 5% CO_2_ and 37 °C in a humidified incubator using RPMI 1640 (Gibco) with 10% (JJ012, SW1353, L2975, HT1080, CH2879 and NDCS1) or 20% (L835, CH3573 and L3252b) heat inactivated foetal bovine serum (FBS) (F7524, Sigma Aldrich). MCS170 was cultured in IMDM (Gibco) with 15% FBS. The authenticity of the cells was confirmed by STR profiling with the GenePrint10 (Promega Benelux BV) and cells were tested for mycoplasma using MycoAlert (Lonza, Switzerland) before the start of the experiments. Cell lines were never cultured for more than three months, and were tested for mycoplasma every 4 weeks (using RT-PCR).

### qRT-PCR

RNA isolation of chondrosarcoma cells and the anonymised controls (growth plate and articular cartilage) was performed using TRIzol (Ambion biosystems, Invitrogen) followed by a standard RNA isolation protocol and cDNA synthesis.^35^ Product size and sequence were validated using Qiaxcel (Qiagen) and Sanger sequencing (Applied Biosystems 48- or 96-cappilary 3730 system, Leiden genome technology centre), respectively. Standard qRT-PCR analyses were performed as described previously^36^ to determine glutaminase, SLC22A1, SLC22A2 and SLC22A3 expression levels. GPR108, CYPa and CPSF6 were used as housekeeping genes for normalisation.^37,38^ Data were normalised using the delta-delta Cq method using Bio-Rad CFX Manager (Bio-Rad).

### Cell viability assay

Cells were counted with the Muse Cell Analyzer (Millipore BV) using the Muse calibration kit (Millipore BV) according to manufacturer’s instruction. Plating was done in densities optimised for each cell line and condition i.e., 3000–15,000 cells per well for 72 h, 200–400 cells per well for one week incubation in triplicates. CB-839 (s7655, Selleckchem), metformin hydrochloride (215169110, Bioconnect), phenformin hydrochloride (219590, Santa Cruz Biotechnology) and chloroquine diphosphate salt (c6628, Sigma Aldrich) were added after the cells adhered overnight. The metabolic drugs were incubated for 72 h or 1 week after which cell viability was measured using the PrestoBlue Cell Viability Reagent (Promega Benelux BV) according to the manufacturer’s instructions. Colourimetric values in the plates were subsequently measured using a Wallac 1420 VICTOR2 (Perkin Elmer). Data were analysed in Graphpad Prism 5.0 (www.graphpad.com). For the combination of metformin, phenformin and chloroquine with AGI-5198 (14624, Life technologies), cells were pretreated for 72 h with AGI-5198 (1 and 10 µM) or DMSO. For the analyses in which the effect of FBS on CB-839 sensitivity was evaluated, the medium with or without FBS and the corresponding concentrations of the metabolic compounds were added at the same time, so after the cells were allowed to adhere overnight.

### Cell count assay

As the PrestoBlue assay measures mitochondrial activity, we confirmed that the effects of metformin, phenformin and chloroquine on cell viability were caused by an absolute decrease in cell number by fixing the cells in 4% paraformaldehyde for 15 min, followed by nuclear staining using Hoechst 33342 (Fischer Scientific). The plates were imaged using a BD Pathway 855 imager (Becton Dickinson), after which the images were processed using an Image-Pro Analyser 7.0 algorithm. Hoechst area was used as a read out to quantify the amount of cells in each well.

### Analysis of apoptosis

For analysis of apoptosis, the caspase-glo 3/7 assay (Promega) was used according to manufacturer’s instructions. Cells were seeded in white walled 96-wells plates (Corning BV Life Sciences) in densities which resulted in 70% confluence after 24 h as described previously.^39^ HT1080, JJ012, SW1353, NDCS1 and CH2879 cells were treated with their IC_75_ of metformin, phenformin, chloroquine and CB-839 (based on 72 h dose response curves). The concentration of compounds used was 10 mM metformin, 100 μM phenformin, 50 μM chloroquine and 6 μM CB-839 if IC_75_, were above these concentrations. CH2879 cells treated with 1 μM doxorubicin (obtained from the in-house hospital pharmacy) and 50 μM ABT-737 (Catalog No. S1002, Selleckchem) were used as positive control. For the negative control doxorubicin and ABT-737 were combined with Z-vad-FMK (550377, BD Biosciences). After 24 h the caspase-glo substrate was added 1:1 followed by incubation of 60 min at room temperature. Wells were analysed using Wallac 1420 VICTOR2. The experiment was performed two times in duplicate. Data was corrected for plane RPMI control and normalised to untreated control for each cell line. Viability was measured on a simultaneously treated plate after 24 h.

### Western blot analysis

HT1080, JJ012, SW1353, NDCS1 and CH2879 cells are treated with their half maximal inhibitory concentration (IC_50_) values (based on dose response curves of 72 h) of metformin, phenformin, chloroquine or CB-839 and lysed after 72 h. A maximum concentration of compounds of 10 mM metformin, 100 μM phenformin and 6 μM CB-839 was used if the IC_50_ was above these concentrations. Western blotting was performed for LC3B (1:1000, clone D11, #3868, Cell Signaling Technology) and phospho-S6 (1:1000, 2F9, #4856, Cell Signaling Technology). As a loading control, α-tubulin (1:10,000, clone DM1A, Sigma-Aldrich Chemie) expression was used. Cells were lysed using hot-SDS buffer (1% SDS, 10 mM Tris/EDTA with complete inhibitor and phosSTOP). For each sample, 10 μg protein was loaded on TGX Stain-Free™ FastCast™ 12% Acrylamide Gels (Bio-Rad). Proteins were transferred to a Polyvinylidene difluoride (PVDF) membrane using Trans-Blot® Turbo™ Transfer System (Bio-Rad) and Trans-Blot® Turbo™ RTA Transfer kit PVDF (Bio-Rad) and detected using enhanced chemo-luminescence (Pierce ECL Western Blotting Substrate Fisher Scientific), followed by exposure of 30 s to 5 min and development of the film (ECL hyperfilm, Amersham, GE Healthcare).

### Cell line metabolic profiling

A Seahorse XFe 96 analyser (Seahorse Bioscience, Agilent) was used to measure the oxygen consumption rate (OCR) and the extracellular acidification rate (ECAR) in chondrosarcoma cell lines JJ012, SW1353 and CH2879 after metformin treatment. Thirty hours prior to the assay, cells were plated in optimised densities being 15,000, 13,000 and 30,000 for JJ012, SW1353 and CH2879, respectively. After 6 h cells were treated with 5 mM metformin for 24 h. Before the measurement, cells were incubated for 1 h in glucose-free RPMI-1640 supplemented with 5% FBS. During the assay, sequential injections of 10 mM glucose (Sigma-Aldrich), 2.0 µM oligomycin A, 2 µM carbonyl cyanide-4-(trifluoromethoxy)phenylhydrazone (FCCP) and 0.5 µM 1:1 rotenone: antimycin A (Cayman Chemicals) established the metabolic profile of all cell lines. Data was normalised to cell numbers measured in each individual well, determined using a Cellomics HCS fluorescent microscope (Thermoe Fisher) after fixation and Hoechst staining. Data represented as the average ± SD of triplicate measurements for metformin treated cells and 5–7 replicates for controls.

## Results

### Glutaminase is a potential therapeutic target in a subset of chondrosarcomas

**Fig. 2:**
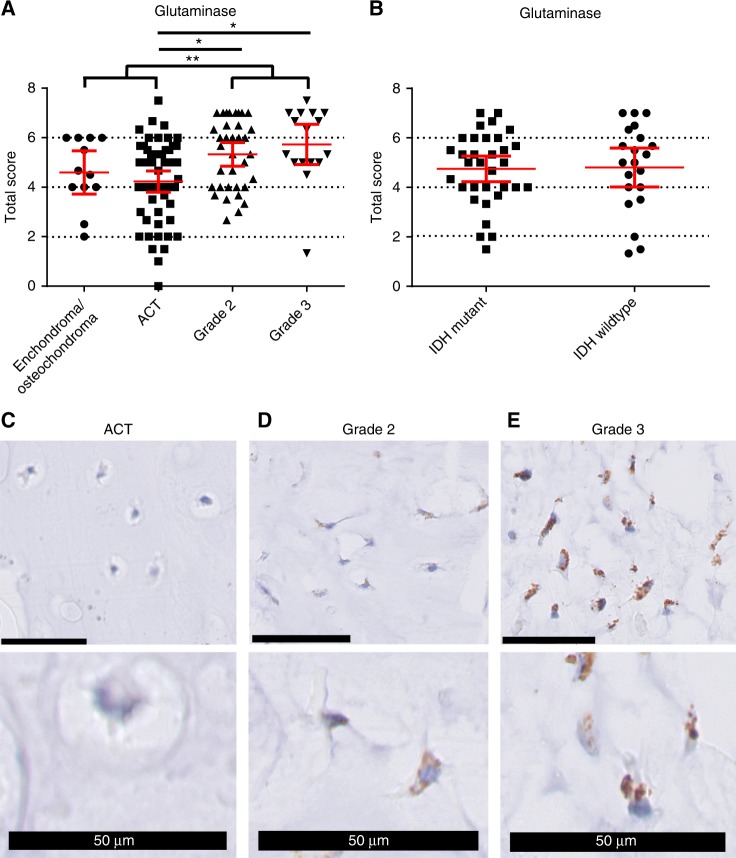
Glutaminase expression correlates to tumour grade but not to *IDH1/2* mutation status. **a** Total score (intensity + percentage) of glutaminase expression. *p = 0.001 by one-way ANOVA with the LSD post-hoc analysis. **p < 0.0001 by independent-samples *T* test, grouping the high-grade and the low-grade cartilage tumours. **b** No difference between *IDH1/2* mutant and *IDH1/2* wildtype central tumours was observed. **c** ACT without expression of GLS, scored as percentage 0, intensity 0. **d** Grade II chondrosarcoma with medium expression of GLS, scored as percentage 2, intensity 2. **e** Grade III chondrosarcoma with high expression of GLS, percentage 4 intensity 3. Black bars represent 50 µm

By immunohistochemistry, a difference in glutaminase expression levels between the different tumour grades was identified (ANOVA, *p* = 0.001), and the post-hoc analysis revealed that specifically the grade II tumours and the grade III tumours had higher expression levels compared to the ACTs (both *p* = 0.001) (Fig. 2a-e). As no difference between central and peripheral cartilage tumours was observed, these were combined in the analyses. Grouping the high-grade cartilage tumours (grade II and grade III chondrosarcomas) and the low-grade cartilage tumours (ACTs and enchondromas/osteochondromas) further demonstrated the significant difference in glutaminase expression levels between high-grade and low-grade cartilage tumours (*p* < 0.0001, independent-samples *T* test) (Fig. 2a). No difference in glutaminase expression between *IDH1/2* mutant and *IDH1/2* wildtype central cartilage tumours was observed (Fig. 2b). Therefore, glutaminase is higher expressed in high-grade compared to low-grade cartilage tumours but does not correlate to *IDH1/2* mutation status.

**Fig. 3:**
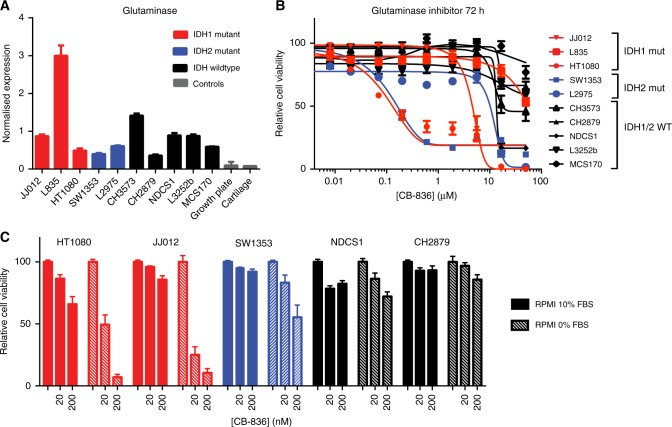
The glutaminase inhibitor CB-839 inhibits chondrosarcoma cell viability. **a** Expression levels of glutaminase in ten chondrosarcoma cell lines and two controls. Glutaminase is higher expressed in chondrosarcoma cell lines compared to growth plate and articular cartilage. **b** Ten chondrosarcoma cell lines were treated for 72 h with CB-839, a glutaminase inhibitor. Sensitivity differed between the different cell lines. **c** Indicated cell lines were treated with 0, 20 or 200 nM CB-839 in the presence or absence of FBS. In the absence of FBS, all cell lines are more sensitive for inhibition with CB-839, especially the *IDH1/2* mutant cell lines

Using qRT-PCR analyses, we demonstrate that all cell lines have higher expression levels of glutaminase compared to the controls (growth plate and cartilage), although expression levels are variable (Fig. 3a). Inhibition of glutaminase using CB-839 in ten chondrosarcoma cell lines revealed that HT1080 (*IDH1*^R132C^), SW1353 (*IDH2*^R172S^) and, to a lesser extent, JJ012 (*IDH1*^R132G^), were very sensitive for glutaminase inhibition, with IC_50_ values below 5 μM (Fig. 3b, Table 1). L2975 (*IDH2*^R172W^), NDCS1 (*IDH1/2*^WT^) and CH3573 (*IDH1/2*^WT^) had IC_50_ values of 10.2, 13.5 and 17.5 μM, while the remaining four cell lines (one *IDH1*^R132C^, three *IDH1/2*^WT^) had IC_50_ values above 50 μM. Interestingly, absence of FBS, increased the sensitivity to CB-839 especially in the *IDH1/2* mutant cell lines, while there was no clear difference in the cell lines with wildtype *IDH1/2* (Fig. 3c). In conclusion, these experiments demonstrate that a subset of chondrosarcoma cell lines is dependent on glutaminase-mediated glutaminolysis to maintain cell viability.

**Table 1:** Absolute IC_50_ values of CB-839, metformin, phenformin and chloroquine upon 72 h of treatment, as determined by PrestoBlue Cell Viability

Compound/cell line	CB-839 (μM)	Metformin (mM)	Phenformin (μM)	Chloroquine (μM)	*IDH1/2* status	Reference
HT1080	0.1	1.20	17.1	19.5	*IDH1* p.Arg132Cys	Rasheed et al.^30^
JJ012	4.6	19.0	504.1	27.1	*IDH1* p.Arg132Gly	Scully et al.^28^
L835	>50	>20	870.3	31.1	*IDH1* p.Arg132Cys	Van Oosterwijk et al.^29^
SW1353	0.2	8.26	74.5	24.6	*IDH2* p.Arg172Ser	ATCC
L2975	10.2	10.6	116.6	30.5	*IDH2* p.Arg172Trp	Van Oosterwijk et al.^29^
CH2879	>50	10.9	193.7	57.9	WT	Gil Benso et al.^31^
NDCS1	13.5	18.1	187.9	16.7	WT	Kudo et al.^34^
CH3573	17.5	>20	343.4	34.0	WT	Calabuig-Farinas et al.^33^
L3252b	>50	16.7	>1000	39.4	WT	Van Oosterwijk et al.^29^
MCS170	>50	10.5	294.7	14.4	WT	De Jong et al.^32^

### Metformin, phenformin and chloroquine inhibit chondrosarcoma cell viability

**Fig. 4:**
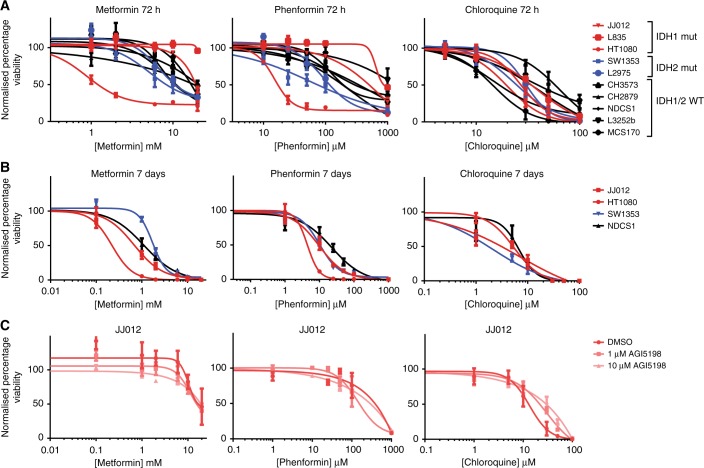
Chondrosarcoma cell lines are sensitive for metformin, phenformin and chloroquine, irrespective of the *IDH1/2* mutation. **a** Chondrosarcoma cell lines were treated for 72 h with corresponding compounds. Cell viability was measured using the PrestoBlue assay. **b** Four chondrosarcoma cell lines were treated for 7 days with the corresponding inhibitors. **c** *IDH1* mutant JJ012 cells were pretreated for 72 h with 10 µM AGI-5198, 1 µM AGI-5198 or DMSO, after which they were treated with a combination of AGI-5198 and the corresponding compounds for 72 h. No effect of AGI-5198 was observed

Treating the chondrosarcoma cell line panel for 72 h with metformin, phenformin or chloroquine demonstrated that sensitivity for these compounds differed between the different chondrosarcoma cell lines (Fig. 4a). With an IC_50_ of 1.20 mM and 17.1 μM after 72 h of treatment, HT1080 cells have a higher sensitivity for respectively metformin and phenformin compared to the other cell lines (Table 1). Treating the chondrosarcoma cell lines for 7 days increased the effect of metformin, phenformin and chloroquine on cell viability (Fig. 4b). Hoechst quantification confirmed that the effects on cell viability were caused by an absolute decrease in cell amount (Supplementary Figure 1). No difference between *IDH1/2* mutant and *IDH1/2* wildtype chondrosarcoma cell lines in sensitivity for metformin, phenformin and chloroquine was observed. To further demonstrate that the IDH1 mutant enzyme does not influence sensitivity to these compounds, the inhibitors were combined with AGI-5198, a specific inhibitor of the mutant IDH1 enzyme, in *IDH1* mutant JJ012 and HT1080 cells. Cell viability (Fig. 4c and Supplementary Figure 2) was unaffected by cotreatment with AGI-5198. These results demonstrate that chondrosarcoma cell lines can be targeted by metformin, phenformin and chloroquine and further demonstrates the dependency of chondrosarcoma cell lines on glutaminolysis independent of the presence of the IDH1/2 mutant enzyme.

### Cellular effects of glutaminolysis inhibition

**Fig. 5:**
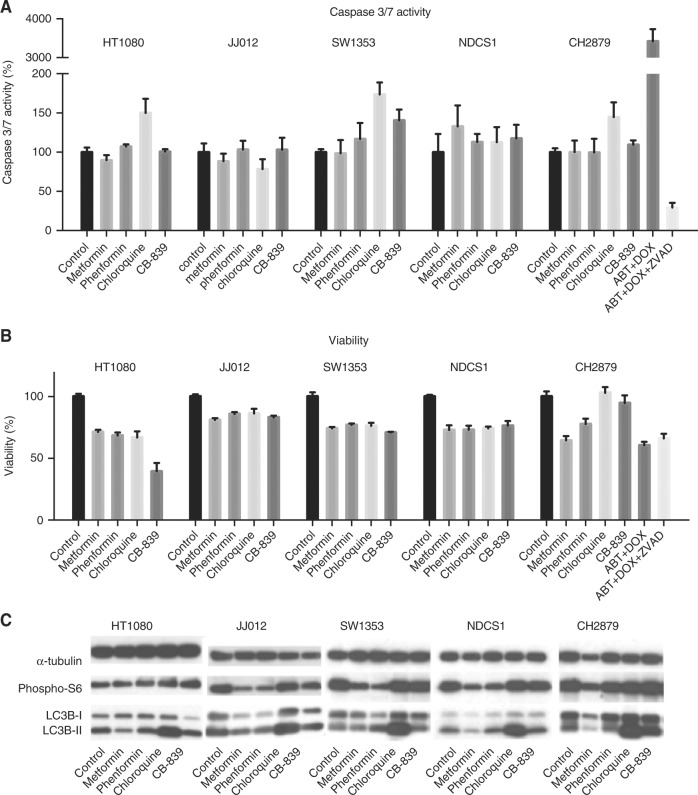
The different compounds provoke different intracellular responses. **a** Caspase 3/7 activity of HT1080, JJ012, SW1353, NDCS1 and CH2879 cells after 24 h treatment with the metformin, phenformin, chloroquine and CB-839, as determined by caspase-glo 7/3 assays. Only chloroquine slightly increases Caspase 3/7 activity in 3/5 cell lines. CH2879 cells treated with ABT-737 and doxorubicin were used as positive control. For the negative control these compounds were combined with Z-vad-FMK. **b** Simultaneously to the measurement of caspase 3/7 activity, cell viability was measured using a Presto-Blue assay. **c** Western blot to evaluate the effect of metformin, phenformin, chloroquine and CB-839 on phosphorylated S6 and LC3B levels in five chondrosarcoma cell lines. Cell lines were treated for 72 h with their corresponding IC_50_ values. Metformin and phenformin decreased levels of phosphorylated S6 in 4/5 and 3/5 cell lines, respectively, and decreased levels of LC3B in 4/5 and 1/5 cell lines, respectively. Chloroquine increased LC3B in all cell lines

To investigate the effect on apoptosis, caspase-glo 3/7 assays were performed. Chloroquine slightly increased caspase 3/7 activity in three out of five cell lines tested (Fig. 5a). While the other compounds did impact cell viability after 24 h (Fig. 5b), no effect on caspase 3/7 activity was observed. Next, we evaluated the effect of the four metabolic compounds on phosphorylated S6 protein levels, as this is an indicator of mTOR activity. As shown in Fig. 5c, metformin and phenformin decreased phosphorylated S6 levels in four out of five and three out of five cell lines respectively. However, metformin and phenformin did not affect phosphorylated S6 levels in HT1080 cells, the cell line that is most sensitive to metformin. Interestingly, metformin decreased LC3B-II levels in four out of five cell lines, which indicates an increase in autophagy.^40^ As expected, chloroquine greatly increased LC3B-II levels. CB-839 did not affect phosphorylated S6 or LC3B protein levels. Thus, while the induction of apoptosis is very limited, metformin and phenformin decreased mTOR activity in chondrosarcoma cells, and metformin decreased autophagy, an effect that is counteracted by chloroquine.

### Metformin is sufficiently transported into chondrosarcoma cells to completely inhibit mitochondrial respiration

**Fig. 6:**
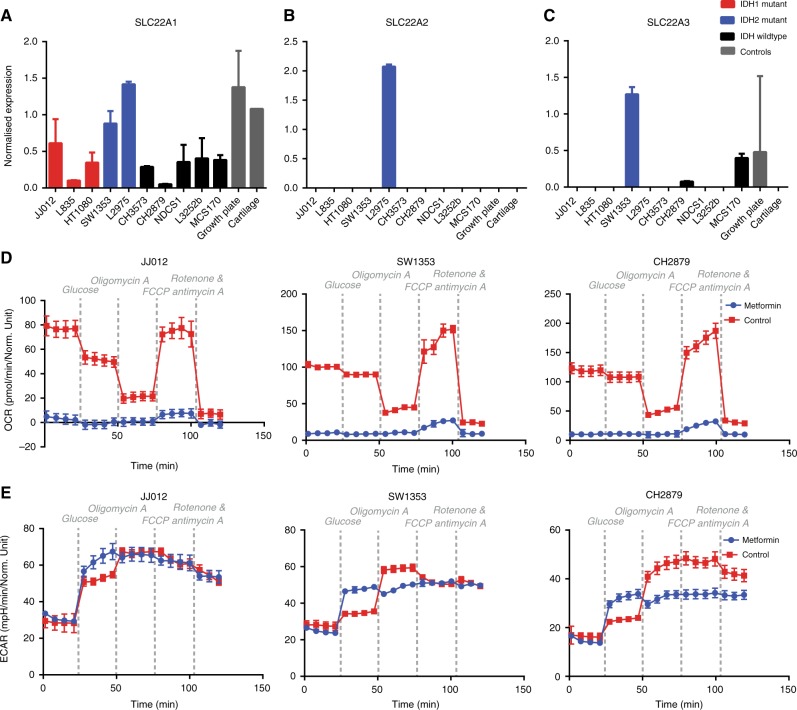
Chondrosarcoma cells sufficiently express SLC22A1 for metformin to completely inhibit mitochondrial respiration. **a** All chondrosarcoma cell lines express SLC22A1. **b** SLC22A2 is only expressed by L2975. **c** Three out of ten chondrosarcoma cell lines express SLC22A3. **d**,** e** The impact of 24 h treatment with 5 mM metformin on the Oxygen Consumption Rate (OCR)(D) and extracellular acidification rate (ECAR)(E) of JJ012, SW1353 and CH2879 was measured by Seahorse experiments. Metformin completely blocks mitochondrial respiration, which is only accompanied by a small increase in glycolysis

Next, expression levels of SLC22A1–3 were determined by qRT-PCR analyses, as these transporters are essential for the cellular uptake of metformin and might explain the variability in sensitivity for metformin. Although expression is variable, all cell lines express SLC22A1 (Fig. 6a). Interestingly, the two *IDH2* mutated cell lines had the highest expression of SLC22A1. SLC22A2 is only expressed by L2975 (Fig. 6b) and SLC22A3 is only expressed by SW1353, MCS170 and CH2879 (Fig. 6c). This demonstrates that all cell lines express transporters for the cellular uptake of metformin, but expression levels differ.

To evaluate the effect of metformin on chondrosarcoma cell metabolism, seahorse experiments with three chondrosarcoma cell lines (one *IDH1* mutated, one *IDH2* mutated and one *IDH* wildtype) were performed. Strikingly, metformin completely inhibited mitochondrial respiration in all cell lines tested independent of *IDH1/2* mutation status or SLC22A1 levels (Fig. 6d). The small increase in OCR observed after the addition of FCCP and increased ECAR levels after glucose injection demonstrate that the metformin treated cells are still viable. Interestingly, metformin treated cells showed higher levels of glycolysis when glucose is present compared to the controls. However, oligomycin A injection increased glycolytic levels in controls but not in treated cells, indicating a maximum in glycolytic energy production is achieved in treated cells after glucose addition (Fig. 6e). The small difference in glycolytic activity between treated and untreated cells cannot compensate for the observed loss in ATP production through mitochondrial respiration, suggesting that chondrosarcoma cells utilise other pathways for energy production.

## Discussion

In this study, we demonstrated a difference in glutaminase expression levels between the different chondrosarcoma grades, with the highest expression observed in high-grade tumours. We therefore examined whether glutaminolysis could be exploited as a therapeutic target for high-grade chondrosarcoma. Based on current ongoing clinical trials targeting glutaminolysis, to which chondrosarcoma patients can be enrolled, we interfered with glutaminolysis in chondrosacoma cell lines at different levels (Fig. 1).

First, we inhibited glutaminase using the glutaminase inhibitor CB-839, and indeed, six out of ten chondrosarcoma cell lines showed IC_50_ values below 20 μM. CB-839 did not induce apoptosis, autophagy or mTOR activity, suggesting that it likely impacts cell viability via other mechanisms.

Second, we used the widely used anti-diabetic drug metformin, which, among other effects, indirectly inhibits glutaminase via c-Myc; (Fig. 1),^21,22^ inhibits complex 1 of the electron transport chain,^20^ and inhibits mTOR signalling.^19^ Indeed, a subset of chondrosarcoma cell lines was sensitive to metformin, especially when treated for a longer time period. mTOR signalling was previously shown to be important in chondrosarcoma^41^ and we confirmed that metformin decreased mTOR activity in all but one chondrosarcoma cell lines tested, which is in line with findings in other studies.^19^ Interestingly, mTOR activity was not inhibited in HT1080 which is the most sensitive for metformin treatment, suggesting that mTOR inhibition alone cannot explain the impact of metformin on chondrosarcoma cell viability. Furthermore, while apoptosis was absent, metformin seemed to induce autophagy in the majority of cell lines tested, which can likely be linked to the effect of metformin on AMPK.^42^ In this study, we further demonstrate that metformin completely blocks mitochondrial respiration in chondrosarcoma cells, likely caused by its effect on complex I of the electron transport chain. However, blocking complex I of the electron transfer chain (and therefore oxidative glutaminolysis) alone was not sufficient to inhibit cell viability, as an even higher concentration of metformin only had a small impact on cell viability within the 24-h time frame of the mitochondrial respiration experiments (Fig. 5b). The small difference in glycolysis observed upon treatment with metformin is likely insufficient to compensate for the total los of ATP production through oxidative means, suggesting that chondrosarcoma cells are not dependent on oxidative metabolism and require other sources, in addition to the increased glycolysis, to supply the necessary energy. In prostate cancer cell lines, it was demonstrated that metformin treatment increased the dependency on reductive glutaminolysis.^43^ Further identifying these pathways might provide interesting targets for combination treatment with metformin.

Third, as an alternative to metformin, we used its lipophilic analogue phenformin. In contrast to metformin, phenformin does not need SLC22A1-3 transport to get into cells.

As expected, the effect of phenformin on cell viability, mTOR activity, and apoptosis was very similar to metformin. Contrary to metformin, phenformin is not used in the clinic due to an increased risk of lactic acidosis. Moreover, we show that all chondrosarcoma cell lines sufficiently express the SLC22A1 transporter that is necessary for metformin uptake, suggesting that there is limited rationale to move to phenformin trials for chondrosarcomas when the metformin trial demonstrates limited efficacy.

Fourth, we evaluated the anti-malaria drug chloroquine, which, in addition to inhibiting glutamate dehydrogenase, is a well-known inhibitor of autophagy and thereby inhibits many other metabolic and signal transduction pathways.^44,45^ The chondrosarcoma cell lines were sensitive to chloroquine. Also, we confirmed that chloroquine inhibited autophagy in all chondrosarcoma cell lines tested. Moreover, a slight induction of apoptosis was seen in three out of five chondrosarcoma cell lines.

Thus, we used four different drugs to evaluate whether the increased dependence on glutaminolysis could be therapeutically exploited using repurposing of existing drugs, and confirmed that a subset of chondrosarcoma cell lines is indeed sensitive to glutaminolysis inhibition. We could not identify a correlation between levels of glutaminase expression and sensitivity for any of these metabolic compounds in the panel of ten chondrosarcoma cell lines. Of note, there was also no correlation of the *IDH1/2* mutation status of chondrosarcoma cells with sensitivity to these compounds, or with the expression levels of glutaminase in primary tumours. We could therefore not confirm the prevailing hypothesis that *IDH1/2* mutant chondrosarcoma cells rely on glutaminolysis to generate sufficient α-KG for *D*-2-HG production,^15–17^ as it seems that also high-grade chondrosarcomas that are wildtype for *IDH1/2* depend on glutaminolysis. To explain this dependency of *IDH1/2* wildtype chondrosarcoma on glutaminolysis, it is tempting to speculate that the hypoxic microenvironment, which is a characteristic of chondrosarcoma,^46^ is equally important or even overrates the effect of the *IDH1/2* mutation in chondrosarcoma. HIF-1α activates pyruvate dehydrogenase kinase, which inactivates pyruvate dehydrogenase, thereby inhibiting the influx of pyruvate into the TCA cycle.^47^ In this context, TCA cycle anaplerosis is required for the synthesis of fatty acids, which is then primarily mediated via glutaminolysis.^48^ Therefore, the hypoxic microenvironment of chondrosarcomas potentially explains why chondrosarcomas depend on glutaminolysis irrespective of the presence or absence of an *IDH1/2* mutation.

Our results indicate that there is limited preclinical rationale to select chondrosarcoma patients for treatment with these compounds based on their *IDH1/2* mutation status. This is in contrast to the studies by Cuyas et al.^16^ and Molenaar et al.,^49^ where, respectively, an increased sensitivity for metformin was identified in an engineered *IDH1* mutant breast cancer cell line, and in an engineered *IDH1* mutant colorectal cancer cell line compared to their wildtype parental cells. The differences in tumour types and the fact that these cell lines harbour an engineered instead of an endogenous *IDH1/2* mutation likely explain the differences in experimental findings.

In conclusion, our results demonstrate that glutaminase is higher expressed in high-grade compared to low-grade chondrosarcomas. High-grade chondrosarcomas are dependent on glutaminolysis which is independent of *IDH1/2* mutation status. This dependence can be therapeutically exploited by repurposing existing drugs that inhibit glutaminolysis, including CB-839, metformin, phenformin and chloroquine.

## Electronic supplementary material

Supplementary Figure 1

Supplementary Figure 2

## Availability of data and materials

The manuscript and the supplementary files contain all potential findings based on raw data analysis. Raw data can be obtained from authors on request.
